# A systematic evaluation of state-of-the-art deconvolution methods in spatial transcriptomics: insights from cardiovascular disease and chronic kidney disease

**DOI:** 10.3389/fbinf.2024.1352594

**Published:** 2024-03-27

**Authors:** Alban Obel Slabowska, Charles Pyke, Henning Hvid, Leon Eyrich Jessen, Simon Baumgart, Vivek Das

**Affiliations:** ^1^ Digital Science and Innovation, Computational Biology—AI and Digital Research, Novo Nordisk A/S, Måløv, Denmark; ^2^ Department of Health Technology, Section for Bioinformatics, Technical University of Denmark, DT U, Kgs Lyngby, Denmark; ^3^ Pathology and Imaging, Global Drug Development, Novo Nordisk A/S, Måløv, Denmark

**Keywords:** spatial transcriptomics, single-cell, deconvolution, Visium, chronic kidney disease, cardiovascular disease

## Abstract

A major challenge in sequencing-based spatial transcriptomics (ST) is resolution limitations. Tissue sections are divided into hundreds of thousands of spots, where each spot invariably contains a mixture of cell types. Methods have been developed to deconvolute the mixed transcriptional signal into its constituents. Although ST is becoming essential for drug discovery, especially in cardiometabolic diseases, to date, no deconvolution benchmark has been performed on these types of tissues and diseases. However, the three methods, Cell2location, RCTD, and spatialDWLS, have previously been shown to perform well in brain tissue and simulated data. Here, we compare these methods to assess the best performance when using human data from cardiovascular disease (CVD) and chronic kidney disease (CKD) from patients in different pathological states, evaluated using expert annotation. In this study, we found that all three methods performed comparably well in deconvoluting verifiable cell types, including smooth muscle cells and macrophages in vascular samples and podocytes in kidney samples. RCTD shows the best performance accuracy scores in CVD samples, while Cell2location, on average, achieved the highest performance across all test experiments. Although all three methods had similar accuracies, Cell2location needed less reference data to converge at the expense of higher computational intensity. Finally, we also report that RCTD has the fastest computational time and the simplest workflow, requiring fewer computational dependencies. In conclusion, we find that each method has particular advantages, and the optimal choice depends on the use case.

## 1 Introduction

The rise and continuous innovation of molecular technologies used within bio-medical research have opened up novel ways of studying diseases and pathogenesis at the cellular level. One such technology is single-cell RNA sequencing (scRNA-seq), which, over the past decade, has become a central tool for studying cellular heterogeneity at the tissue level, thereby gaining crucial mechanistic insights into diseases (Aldridge and Teichmann, 2020). Such insights form the basis for, e.g., early-phase drug target identification. A more recent technology in the biological research toolbox is spatial transcriptomics (ST). ST, in combination with scRNA-seq, can elucidate how gene expression and specific cell types localize spatially in tissues (Moses and Pachter, 2022). Understanding cellular migration is key in inflammation, which is a common disease trait.

**FIGURE 1:**
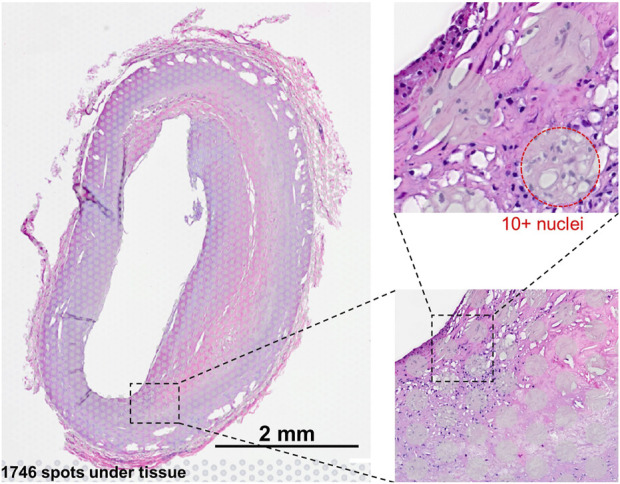
Visium slide showing examples of the barcoded 55-um spots, where each spot represents a mixture of cells, typically between 5–10 cells.

A central limitation of multiple ST technologies, including 10× Visium (10x Genomics), is that the resolution is not at the single-cell level, and even for high-resolution methods, the spatial unit or ‘pixel’ is not guaranteed to align with each individual cell. The transcriptomic profile will, therefore, stem from a mixture of up to approximately 10 cells and often more than one cell type (Figure 1). This challenge has inspired a surge in bioinformatics methods, aiming to split up the location-specific transcriptomic profile into its constituents and assign cell types based on the reference data, i.e., signal deconvolution. Several deconvolution methods for ST data have been published (Li et al., 2022; Yan and Sun, 2023). In many cases, these methods are only validated on healthy brain samples from mice, where cell type populations may be more spatially segregated and well-defined (Dong and Yuan, 2021; Cable et al., 2022; Kleshchevnikov et al., 2022). Here, we seek to validate methods using cardio-renal disease data, which encompass a spectrum of related disorders of the heart, blood circulation, and kidneys in relation to cardiovascular disease (CVD), which is a complex chronic disease accounting for approximately four million deaths every year in Europe alone, corresponding to 45% of all deaths (Townsend et al., 2016). ST has the potential to play a major role in unraveling the underlying mechanisms, but currently, the described challenges with the resolution level are a major hindrance. Currently, ST plays an important role in target validation and identification. This often requires an accurate connection of the gene to the cell type where spatial deconvolution is central. Furthermore, deconvolution allows statistical approaches across multiple tissue sections to distinguish artifacts from robust effects in an automated fashion.

In this study, we set out to evaluate three current state-of-the-art deconvolution methods in the ST signal. We applied the deconvolution methods to Visium ST data from arterial and kidney samples from healthy and pathological states using three different methods, namely, RCTD (Cable et al., 2022), Cell2location (Kleshchevnikov et al., 2022), and spatialDWLS (Dong and Yuan, 2021), all of which have previously been shown to achieve high accuracy in benchmarks (Li et al., 2022; Yan and Sun, 2023). A challenge here is the lack of gold standards and clinically relevant chronic tissue data. To address this, we performed a systematic evaluation using novel data obtained by manual annotations and subsequently validated the *in silico* labels with an expert histopathologist.

## 2 Materials

### 2.1 Arterial plaque and kidney samples

Three publicly available datasets (Wirka et al., 2019; Pan et al., 2020; and Alsaigh et al., 2022) were merged to create an atherosclerosis single-cell RNA (scRNA) atlas. The datasets comprised arterial samples from patients undergoing carotid endarterectomy and heart transplant coronary arteries. The merged atlas consisted of 60,676 cells with nine annotated cell types (Supplementary Figure S1). It was observed that both RCTD and DWLS experienced memory issues using the full data set when running locally. RCTD has a default setting to downsample reference data on a per-cell-type basis, although the choice of sample size is not validated in depth. To accommodate these issues and improve computational time, an analysis was run to investigate the repeatability of deconvolution results with smaller reference sizes (See results Figure 3). Going forward, random sub-sampling was performed to obtain 3,000 cells per cell type.

**FIGURE 2:**
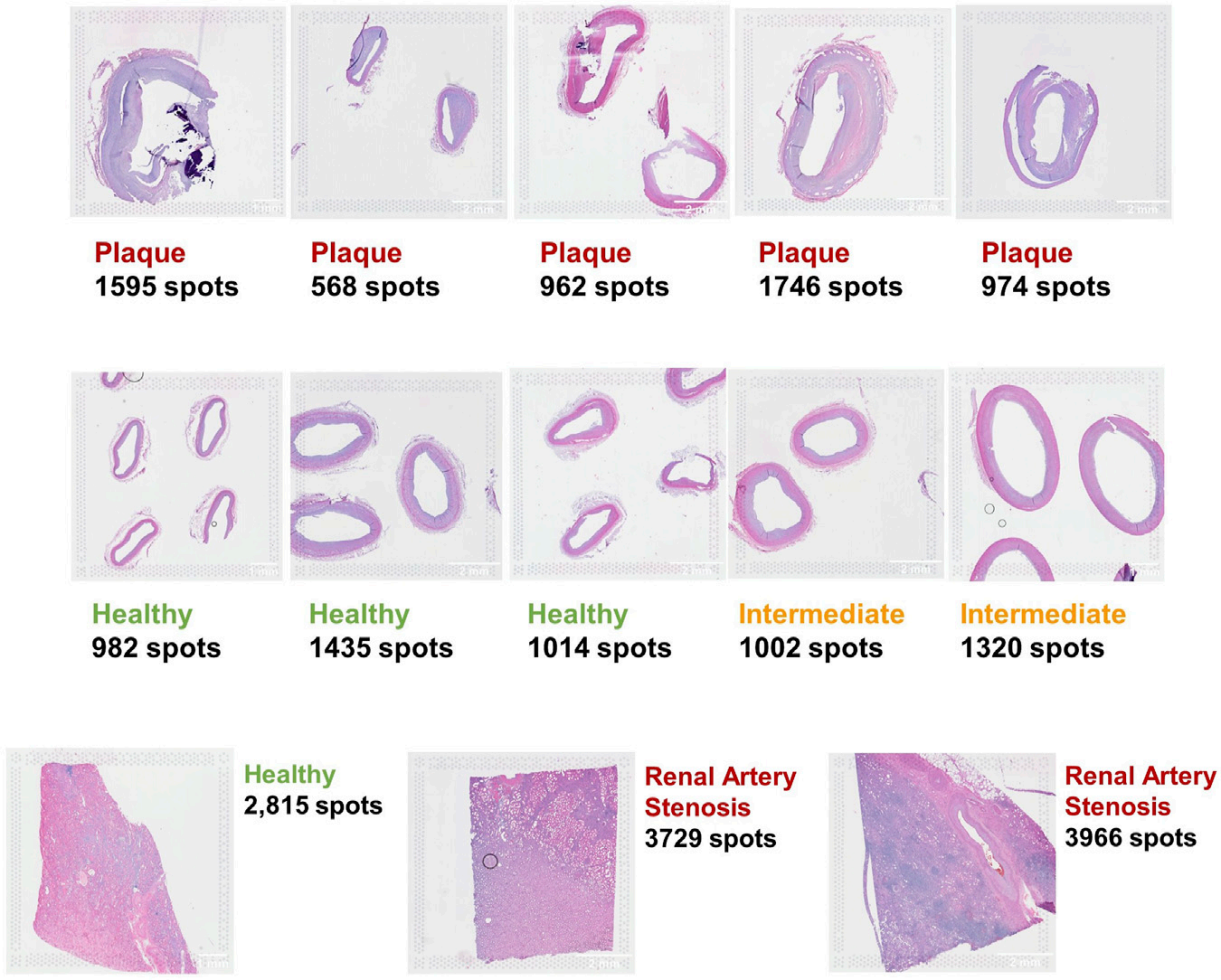
Ten coronary artery FFPE samples (top two rows) and three kidney FFPE samples (bottom row) analyzed with the 10× Visium protocol.

A total of 10 spatial transcriptomics (ST) samples were generated from formalin-fixed and paraffin-embedded (FFPE) samples of coronary arteries isolated from explanted hearts using the 10× Visium protocol (10X Genomics, 2022; 10X Genomics, 2023). Among the 10 ST samples, five were pathological with clear signs of atherosclerosis, two had early signs of plaque formation, and three were healthy without plaque (Figure 2). For each of the 10 samples, near-adjacent tissue sections (from 1 to 4) from the same coronary artery sample were placed in barcoded capture areas and processed. The CVD samples ranged from 568 to 1,746 spots, and the three CKD samples covered up to 3,966 of the 5,000 total available spots for each capture area.

### 2.2 Visium protocol

Tissue sections were placed on Visium slides (1–4 serial sections per sample), stained with hematoxylin and eosin (H&E), and imaged using a VS200 slide scanner (Olympus Life Sciences), prior to de-staining and overnight hybridization with the Visium human version 1 probe set. The following day, the probes that were hybridized to mRNA in the tissue sections were eluted and ligated to spatially coded oligonucleotides on the Visium slide. Based on these, a cDNA library was created for each sample. The libraries were sequenced on a NovaSeq 6000 (Illumina) sequencing platform, according to the manufacturer’s instructions, using a NovaSeq 6000 S2 Reagent Kit v1.5 (Illumina). Subsequently, reads were aligned with their corresponding probe sequences, mapped to the Visium spot where each probe was originally captured, and finally aligned with the original H&E stained image of the tissue section using the software application SpaceRanger version 1.3.0 (10X Genomics, 2022).

### 2.3 Evaluating reference for CVD data

For the evaluation step, a partial ground truth cell type reference was obtained from an experienced histologist with tissue-specific expertise. The ground truth cell type assessment consists of approximately 50 spots per sample, for which it was possible to unambiguously determine a dominant cell type from the available high-resolution bright-field microscopy images. Across 10 samples, 496 spots were labeled as one of the three cell types: smooth muscle cell (SMC), macrophage (Mø/MP), or endothelial cell (EC). These novel data formed the basis of the systematic evaluation and are as close to a golden standard as possible, given the current state of technology.

### 2.4 Evaluating reference for CKD data

For CKD samples, a scRNA reference (Supplementary Figure S1) was obtained from the publicly available Kidney Precision Medicine Project (KPMP) data repository (Hansen et al., 2022). Similar to the atherosclerosis atlas, random subsampling was performed on a per-cell-type basis to reduce the data set size.

## 3 Methods

We have systematically evaluated using three different models, as described below.

### 3.1 Robust cell type decomposition

This model utilizes Poisson distribution to model counts (Cable et al., 2022), as depicted below in Equation 1:

$$Y_{i,j}|\lambda_{i,j}∼Poisson\left(N_{i}\lambda_{i,j}\right)$$ (1)

Equation 1.

Given $\lambda_{i,j}$ , the random variable $Y_{i,j}$ follows a Poisson distribution with the scaling factor $N_{i}$ and rate parameter $\lambda_{i,j}$ , where the rate parameter is modeled around the product of the underlying cell-type counts and the estimated expression signatures obtained from labeled single-cell data.

$$\log\left(\lambda_{i,j}\right)=\alpha_{i}+\log\left(\sum_{k=1}^{K}x_{i,k}\;S_{k,j}\right)+\gamma_{j}+\epsilon_{i,j}$$ (2)

Equation 2.

In Equation 2, $\alpha_{i}$ represents fixed spot-specific effects. $S_{k,j}$ is the signature gene matrix, which, in this case, is the mean gene expression profile per cell type. The parameter $\gamma_{j}$ represents gene-specific random effects arising from varying gene sensitivity between sequencing technologies. This effect is estimated before other parameters by combining the ST data into a bulk measurement and comparing this to the scRNA expression. $\epsilon_{i,j}$ accounts for other random effects or sources of variation, including overdispersion.

### 3.2 Cell2location

This is a Bayesian model for deconvoluting ST data into cell-type absolute abundances (Kleshchevnikov et al., 2022). Cell2location requires two hyper-parameters to be set by the user: the expected number of cells per spot and a regularization parameter. The first was found by inspecting histology images and counting the nuclei for a representative selection of spots and was set to 8 for CVD samples. The regularization parameter represents the degree to which individual spot sensitivity deviates from the mean within the specific experiment, such that a high value signals consistent detection sensitivity. This parameter was kept at the relatively low default value of 20 to account for the varying quality of batches which come from an early exploratory cohort of ST samples.

Cell2location models the observed counts using a negative binomial distribution, as in Equation 3. The rate parameter is again defined as a function of the signature matrix learned from the single-cell reference data and the hidden cell-type counts. Spot- and gene-specific rates are estimated, and parameters are introduced to account for variation in technological sensitivity, shifts due to contaminations, and spot-specific sensitivity.

$$Y_{i,j}∼NB\left(\mu_{i,j},\alpha_{j}\right)$$ (3)

Equation 3.

$$\mu_{i,j}=\left(m_{j}\cdot \sum_{k}x_{i,k}S_{k,j}+s_{j}\right)\cdot \gamma_{i}$$ (4)

Equation 4.

Here, μ is the rate parameter and α accounts for over-dispersion (Eq. 3). The parameter mj accounts for technological variation in the sensitivity to specific genes. The parameter sj describes an absolute shift in RNA-capturing potential gene-specific contaminations. Finally, the scaling parameter γi is included to account for differences in general sensitivity in specific spots (Eq. 4).

### 3.3 Spatial dampened weighted least squares

SpatialDWLS (Dong and Yuan, 2021) is a weighted least squares approach for deconvoluting cell-type proportions of ST spots based on the method DWLS (Tsoucas et al., 2019), previously published for cell-type deconvolution in bulk RNA-seq data.

Similar to other methods, the observed counts are modeled as a product of the estimated expression signatures and the hidden cell-type counts of interest.

$$\hat{S}\hat{x}=Y$$ (5)

Equation 5.

The weighted least squares error minimization problem is defined as follows:

$$\min_{\tilde{x},\tilde{x}>0}\sum_{i=1}^{n}w_{i}{\left(Y_{i}-\sum_{j=1}^{k}{\hat{S}}_{ij}{\tilde{x}}_{j}\right)}^{2}$$ (6)

Equation 6.

The weights, w, are defined as a function of the dependent variable, the estimated cell-type numbers x, and are, therefore, determined through iteration, with the first iteration being unweighted ordinary least squares (OLS). This proceeds until the solution converges. The authors introduce a dampening constant to avoid extreme values of weights. This constant is determined by cross-validation of the signature genes to minimize variance.

### 3.4 Influence of reference sample size on repeatability

The sample CVD2 was used for the analyses in combination with different subset sizes of the Atherosclerosis Atlas. Five different subset sizes, with three different replicates of each size, were generated. Random samples were taken without replacement for each of sizes 30, 100, 400, 1,000, and 3,000 cells per cell type. If a cell type had fewer observations available than the chosen sample size, all observations were kept instead. This was relevant for B cells with a total of 2,607 cells, SMC subtypes with a total of 1,016 cells, and mast cells with a total of 764 cells in the atlas. For each of the random subsets, deconvolution of the CVD2 sample was run, and the results were compared using Spearman’s correlation coefficient as a measure of similarity of the resulting cell-type proportions.

### 3.5 Computational time assessment

For the three methods, the computational time durations of the training steps and deconvoluting steps were recorded individually. For recording the computational time in R, the package tictoc (V.1.1) was used (Izrailev, 2023). In Python, the built-in timing functionality of the Cell2location package was used to record the time at each step. The computer used for all computation time recordings was a Windows 10 pc, with a 2.70 GHz, Intel i7-10850H CPU and 16 GB of RAM. For GPU computation, the NVIDIA Quadro P620, 2GB/512 CUDA core, graphics unit was used.

### 3.6 Evaluation using histologist-provided annotation

The predictive accuracy of each method is estimated by comparing the deconvoluted proportions of select spots with annotations provided by a histologist. The available cell-type annotations are limited to major anatomically and visually distinct cell populations, which include smooth muscle cells (SMCs), endothelial cells (ECs) that line the vessel lumen, and in plaque sample aggregated macrophages (MPs). For comparing the histologist-assessed spots (n = 496), a dominant cell type had to be determined from the proportions obtained in each deconvolution. Each spot was classified as the cell type with the highest predicted proportion, and the smooth muscle cell subtypes were grouped. The accuracy (ACC) was calculated per method corresponding to the proportion of true predictions out of all the predictions included.

For CKD samples, precision and recall were determined, and AUROC was calculated using the pROC package (Robin et al., 2011) based on the histologist’s annotation of podocyte-containing glomeruli. For a given spot to be classified as containing at least one podocyte, a threshold of 15 percent predicted content was used.

## 4 Results

### 4.1 Deconvolution accuracy with variable reference scRNA-seq subsets

**FIGURE 3:**
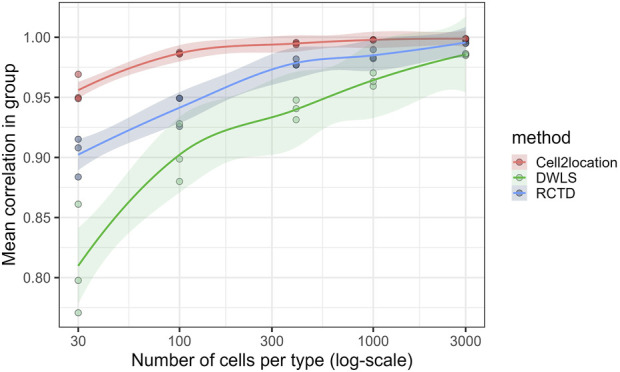
Single spatial sample underwent deconvolution three times at each sample size (30, 100, 300, 1,000, and 3,000 cells/cell type) using independent subsets. Each point is the correlation (Pearson) between predicted cell-type proportions of two separate deconvolutions using independent, random sub-samplings, at the given number of cell per cell type. Cell2location achieves noticeably consistent results at as little as 30 instances of each cell type. LOESS regression with 0.95 confidence interval has been applied for each method.

The effect of reducing the reference size on the results of deconvolution was investigated by subsetting the scRNA data. To assess the confidence with which each method performs deconvolution at a given reference size, a number of replicate analyses were run. In each group of three subsets, the three possible pairwise Pearson correlations were calculated. It was observed that the correlation rose from around 0.8 for spatialDWLS, 0.9 for RCTD, and 0.95 for Cell2location, for subsets of 30 cells/cell type, up to at least 0.95 for all three methods at a subset of 3,000 cells/cell type (Figure 3). Correlation was similarly calculated on a per-cell-type basis. Mast cells, SMC subtypes, and NK-/T-cell estimates generally displayed the lowest correlation within each group, indicating some uncertainty of the results when the reference sample size was too small. All cell types, except mast cells and SMC subtypes for spatialDWLS, achieved coefficients above 0.90 at the largest sample size. The macrophage/monocyte group was consistently the highest correlating cell type between the replicate runs for all three methods (Supplementary Figure S2).

### 4.2 Evaluating method accuracy for major cell types

All three methods performed poorly on the observed endothelial cells (ECs). Only DWLS predicted any of the assessed spots as EC-dominant, but none were in agreement with the ground truth (Table 1). The resulting accuracy scores indicate a similar performance for all three methods. RCTD achieved a slightly higher score than the other two methods at 0.734, compared to 0.702 and 0.708 for Cell2location and DWLS, respectively, corresponding to 13–15 additional true predictions. The standard deviation of the accuracy calculated across the 10 samples is too high to distinguish the methods meaningfully. Most endothelial cells were predicted as smooth muscle cells across all methods. Additionally, all three methods predicted a few smooth muscle cell spots as fibroblast-dominant, and between 17 and 29, macrophage spots were predicted as smooth muscle cells. Lastly, Cell2location assigned eight macrophages as NK/T-cells.

**TABLE 1:** Accuracy for each method was calculated as the rate of success for predicting major cell types in spots with expert-supplied ground truth. Standard deviation (SD) values across the 10 samples are shown in parentheses.

	Cell2location			RCTD			DWLS		
True type	Predicted type			Predicted type			Predicted type		
	EC	MP	SMC	EC	MP	SMC	EC	MP	SMC
EC	**0**	12	94	**0**	11	96	**0**	7	99
MP	0	**49**	17	0	**54**	20	0	**45**	29
SMC	0	3	**299**	0	3	**310**	2	4	**306**
**ACC**	**0.702 (SD** ± **0.119)**			**0.734 (SD** ± **0.075)**			**0.708 (SD** ± **0.065)**		

### 4.3 Cell-type-dependent inter-method agreement assessment

**FIGURE 4:**
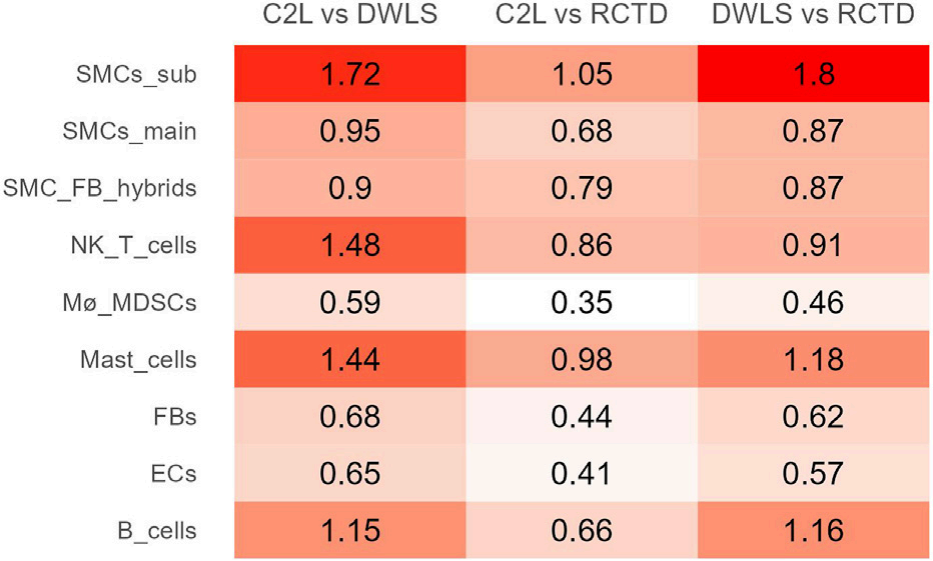
RMSRD between the methods for each cell-type group. The smaller values in C2L vs. RCTD signify more similar predictions by these methods.

Root-mean-square relative difference (RMSRD), a metric for determining the pairwise difference between two methods on a per-cell-type basis, was calculated for each pair of methods. A smaller value signifies a smaller mean difference between the two methods in relation to the mean proportion of the cell type. A generally greater disagreement is observed for subtype SMCs, NK/T, mast, and B cells, particularly when compared to DWLS (Figure 4; Supplementary Table S1).

For kidney samples, the cell types subject to major variability between methods include thin descending limb (Thin_DL) cells, parietal epithelial cells (PECs), connecting tubule (CT) cells, and ascending thick/thin limb (ATL_TAL) cells. Conversely, the rare cell type podocyte (POD) had a high agreement between the methods, as did endothelial cells (ECs), distal convoluted tubule (DCT) cells, and proximal tubule (PT) cells. The previous pattern of a higher agreement between Cell2location and RCTD does not seem to be repeated for these samples (Supplementary Figure S3).

The high agreement between the methods on the podocyte population is of particular interest as it is the most sparse cell type in the reference data (n = 244). In comparison, PECs are also rare (n = 631) but are subject to a much higher method-based variation.

### 4.4 Differences in computational time

For each of the three methods, computational times were recorded for a number of samples of varying spot-counts and for the different reference subset sizes used. The time spent was recorded individually for any data preparation and then for the deconvolution itself. The data preparation step was defined as all actions that do not need repeating after the ST sample is deconvoluted but can be applied directly to the next sample. All three models were found to have a strong linear fit between time spent and the number of spots for deconvolution or the number of cells for preparation (Supplementary Figure S4).

RCTD and spatialDWLS had similar deconvolution times in the 2–15 min range for ST samples, with up to 3,000 spots. RCTD completed preparations faster, rarely needing more than 5 min. Cell2location was much slower, spending up to 65 min on estimating the expression profiles in a 32,000-cell scRNA subset. When deconvolution was carried out using Cell2location, the timing ranged from 35 min to 2.5 h per sample.

### 4.5 Evaluating kidney samples

Given the limited kidney ST samples, it was not possible to perform all identical experiments as for CVD. However, a few experiments were performed to assess the performance of deconvolution. Across three kidney samples, 110 spots were labeled as podocyte-containing glomeruli by expert histologist evaluation of microscopy images, as shown in Supplementary Table S2. Under the assumption that this evaluation provides a complete ground truth of podocyte locations, the performance metrics were calculated across all spots, as represented in Table 2. A 10% estimated proportion threshold has been used to classify a spot with a podocyte designation. Cell2location seemed to have a higher precision value compared to RCTD and DWLS, while all three methods have similar AUROC values.

**TABLE 2:** Precision and recall values based on podocyte predictions for each method, as well as AUROC value for the podocyte prediction classification threshold using pROC.

	Precision	Recall	AUROC
Cell2location	0.6	0.74	0.98
RCTD	0.36	0.83	0.98
DWLS	0.38	0.86	0.96

## 5 Discussion and conclusion

The aim of this study was to systematically implement and evaluate a selection of deconvolution methods for internally produced ST data within Novo Nordisk A/S. With a specific focus on human cardio-renal samples in various disease stages, for which limited data have been published, we consulted existing benchmarks to narrow down the large number of published deconvolution methods, like B Li et al., Yan et al., and Sangaram et al. (2023). All three benchmarked studies highlighted the methods Cell2location, RCTD, and spatialDWLS as the most accurate and top ranked out of all the deconvolution methods evaluated. These results additionally have been highlighted by 10× Genomics guides for use with Visium data (https://www.10xgenomics.com/analysis-guides/benchmarking-methods-to-integrate-spatial-and-single-cell-transcriptomics-data). Computational pipelines were created for the three methods RCTD, Cell2location, and DWLS. Based on scRNA-seq atlas, the expression profiles for annotated cell types were estimated, following the recommended guidelines for each method. These profiles were then used with their respective methods to deconvolute 10 ST samples of arteries and three ST samples of kidney tissue at various stages of the disease.

In practice, the number of cells necessary to establish a representative cell type profile will vary depending on the heterogeneity of the cell type in question, as well as the quality of the labeling within the reference data. If a cell type is highly homogeneous in the context of the tissue studied and the instances of this cell type within the reference are accurately labeled, one might expect that a very small number of cells would be sufficient to estimate the expression profile. Conversely, if the cell type is highly heterogeneous, e.g., with multiple unlabeled sub-populations, a larger sample size of representative cells would be preferable to capture as much variance as possible. Despite this, all three models reduce the reference data to a single expression profile for each cell-type label, and so, for highly diverse cell types, minority sub-populations might be drowned out by the averaging of signals. This is an argument for careful cell-type labeling prior to deconvolution and for dividing cell-type labels into subgroups, especially for diverse groups. In addition, this illustrates the challenge of approaching a cell type definition as a static expression profile as, clearly, this will be context-dependent. Thereby, one well-defined cell type may exhibit a multitude of expression profiles.

The variations in cell type estimates from replicate runs were evaluated to validate the choice of subsampling. Most estimates were seen to converge with high uniformity between repeat runs as the subsampling neared 1,000–3,000 cells per cell type. Nonetheless, Cell2location achieved higher consistency at lower reference data sizes than both RCTD and DWLS, where DWLS struggled with the repeatability of specific cell types even at high counts.

It should be noted that since publicly available single-cell atlases were used, we would expect the data to be widely representative for the given tissue. Nonetheless, it might prove beneficial to use reference data generated from the same tissue samples, as used for the ST protocol. Therefore, it is also valuable to investigate in future how much single-cell or nucleus data is necessary to be generated to establish a sufficient reference.

Cell2location and RCTD are both probabilistic methods that rely on discrete probability distributions to model read counts. Cell2location additionally uses a Bayesian probabilistic programming approach. This resulted in remarkably longer computation times observed despite GPU acceleration, but it also provided a probabilistic result in the form of a distribution for every estimated cell-type proportion, allowing the extraction of cell-type abundances at a chosen confidence level. A benchmark was established to compare the predictive accuracy. Results indicated that all methods performed well in predicting cell-type distributions, at least as far as common cell types were concerned. ECs of the vessel lumen were a problem for all three methods, but it is important to note that the Visium spots are much wider than the endothelial monolayer. A dominant EC signal can, therefore, not be expected in these spots.

Thus, the three methods for deconvolution were successfully applied to internally generated cardio-renal disease data, using the 10× Visium protocol. Ten coronary artery spatial transcriptomics samples were deconvoluted, and all major cell types, including smooth muscle cells, fibroblasts, and macrophages, were observed to localize in the expected anatomical regions of the arterial vessel. An agreement between the methods was found to be high for macrophages, a major cell type of interest in cardiovascular disease.

With ground truth labels supplied by an experienced histology expert, additional method accuracies were evaluated. All three methods achieved similar accuracy, with RCTD outperforming the others by a small margin. Cell2location was found to require the least amount of reference data for the supervised step to achieve a consistent output, requiring as little as 100 reference cells per cell type for convergent results. Following this analysis, the same workflow was applied to three spatial transcriptomics samples at varying stages of CKD. However, due to a smaller number of ST samples available, similar inferences could not be made. This could be considered a limitation to the study, given the samples available in the CKD. However, we tried to make a sanity assessment in a quantitative manner with the limited CKD samples available. We could observe a similar method agreement, and podocytes were identified as a cell type of interest for further analysis. Due to its well-segregated localization in glomeruli only, all three methods offered decent precision metrics of convergence between the model and the histopathologist’s expert assessment. Taken together, we think that all three methods are capable of deconvoluting verifiable cell types based on the assessments performed in this brief systematic assessment exercise. It will be interesting to see how these performances will eventually hold or evolve with emerging single-cell disease atlases and spatial atlases in cardio-renal areas from public and private consortium initiatives that can also eventually pave a way for disease understanding, drug target discovery, and validation. Our current scope of study is also limited to only cardio-renal datasets; hence, the generalizability to other cardiometabolic organs impacted in chronic cardiometabolic diseases needs to be explored. In the current study, we are only evaluating a single spatial technology; however, in future, we aim to address these limitations across tissues or organs and validate them in more specific high-resolution platforms, e.g., Xenium ST or Visium HD, when available to improve our *in silico* drug target identification and the validation process. We also encourage the community to perform similar assessment initiatives for chronic human tissues with disease stages where limited data are available when such large observational cohorts are available at the atlas level since this will guide the scientific community not only about the tool’s performance but also where some such spatial technologies are limited, and eventually, future high-resolution ST technologies will pave the way.

Although it has long been possible to identify cell types of interest by morphology or by targeting a few well-known marker genes or proteins, the newer, untargeted ST technologies may be able to provide much higher granularity in the cell type and subtype identification. The ability to produce data with near whole-transcriptome coverage and single-cell resolution through deconvolution is a very powerful method that provides a more unbiased and systematic way of studying tissue organization at different disease states and identifying cell types of interest. In summary, we found RCTD to be useful in terms of accuracy, computational speed, and ease of implementation. Cell2location may be highlighted for its ability to achieve robust results with limited reference data, which is an important consideration during the experimental design phase. Additionally, Cell2location allows for more extensive probabilistic modeling due to its Bayesian framework. Therefore, Cell2location may be recommended for more extensive workflows and in-depth modeling where computational time is not a concern.

## Data Availability

The datasets presented in this study can be found in online repositories. The names of the repository/repositories and accession number(s) can be found in the article/Supplementary Material. Danish legislation regarding sharing of human data requires that the spatial transcriptomics data can be made available upon reasonable request to the corresponding author and approval from The Danish Data Protection Agency. Scripts for running deconvolutions and output can be found on GitHub (https://github.com/albanobel/Deconvolution-for-ST-in-Cardiorenal-Disease.

## Supplementary material

SUPPLEMENTARY FIGURE S1 Single cell RNA reference data UMAP with cell type labels. Top are major CVD cell types, bottom are major cell types within CKD atlas. CKD abbreviations as follows: PT, Proximal Tubular; EC, Endothelial Cell; TAL, Thick Ascending Limb; ATL, Ascending Thin Limb; IC, Intercalated Cell; CNT, Connecting Tubule; DTL, Descending Thin Limb; DCT, Distal Convoluted Tubule; PEC, Parietal Epithelial Cell; POD, Podocyte; PC, Principal Cell.

SUPPLEMENTARY FIGURE S2 For each method deconvolution of sample CVD2 was repeated with multiple different reference sizes. Correlation between independent deconvolutions was calculated to determine degree of variation in prediction as a function of reference data size. Cell2location is observed to be more consistent across cell types.

SUPPLEMENTARY FIGURE S3 The RMSRD between each pair of methods, with a lower value indication more similar results for the specific cell type.

SUPPLEMENTARY FIGURE S4 Benchmarking the deconvolution time requirements across methods. For the reference preparation step the x-axis indicates number of cells in single cell reference dataset. For deconvolution, x-axis indicates number of spots subject to deconvolution.

SUPPLEMENTARY TABLE S1 RMSRD is calculated per sample and the mean and sd is presented instead of the overall value for clarification supporting Figure 4 around inter-method agreement assessment in CVD samples.

SUPPLEMENTARY TABLE S2 True and false predictions by each method were determined allowing for precision, recall and AUROC metrics to be calculated.

SUPPLEMENTARY TABLE S3 Represents the markers used for deconvolution by all 3 methods in CVD dataset. For RCTD and spatialDWLS the top 10 marker genes per cell type are shown as determined by the supervised step of the methods. RCTD and spatialDWLS have similar log-fold-change based approaches to marker selection. For Cell2location the genes with the highest estimated expression per cell type are shown. Cell2location estimates expression of all genes for each cell type but does not directly consider differential expression at the supervised step.
