# Supplementary figures and images for: A systematic evaluation of state-of-the-art deconvolution methods in spatial transcriptomics: insights from cardiovascular disease and chronic kidney disease
Source: Front Bioinform. 2024 Mar 27;4:1352594. doi: 10.3389/fbinf.2024.1352594 (PMC11004278; doi:10.3389/fbinf.2024.1352594)

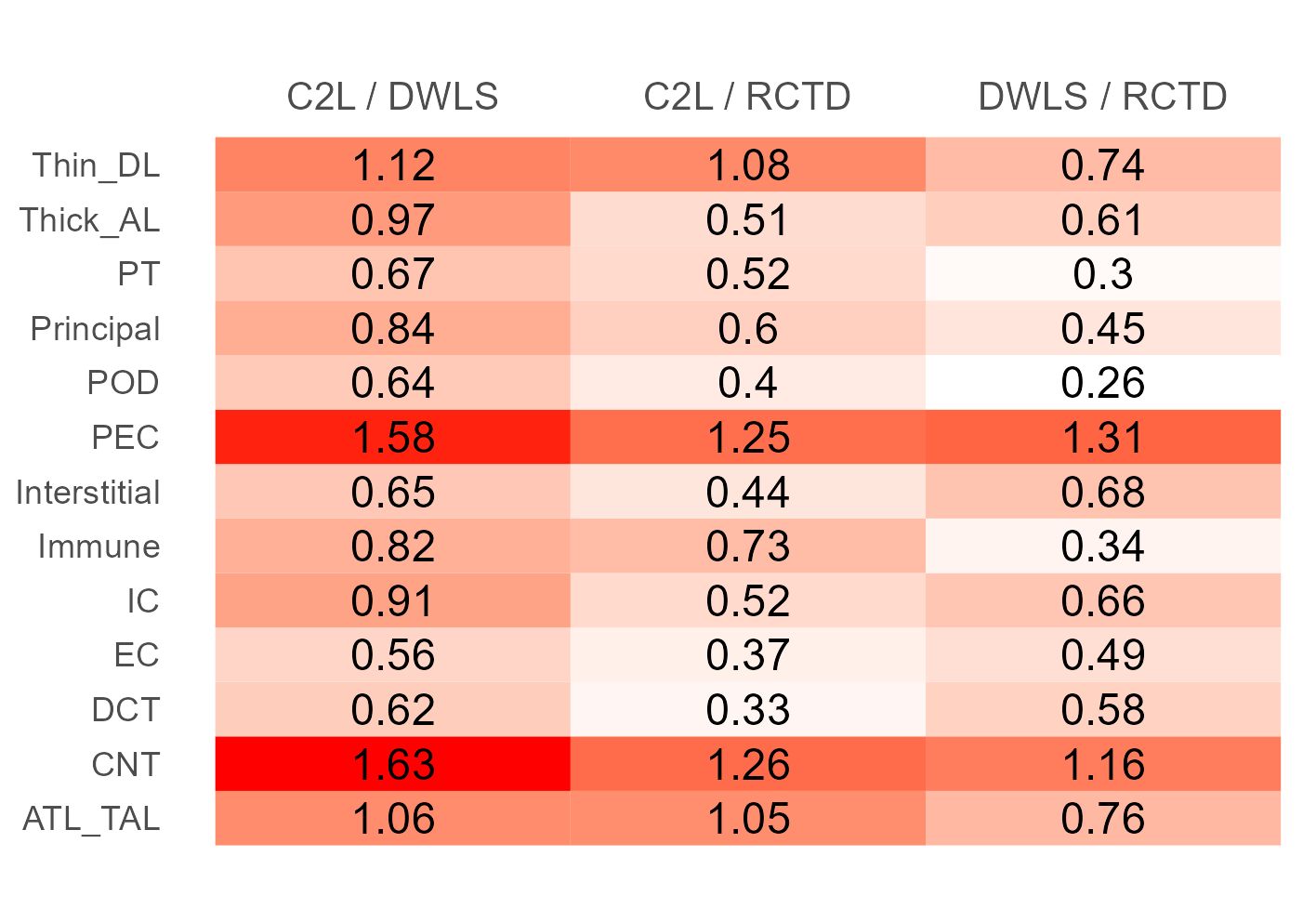

Supplement: Supplementary file 2 [file Image3.JPEG]

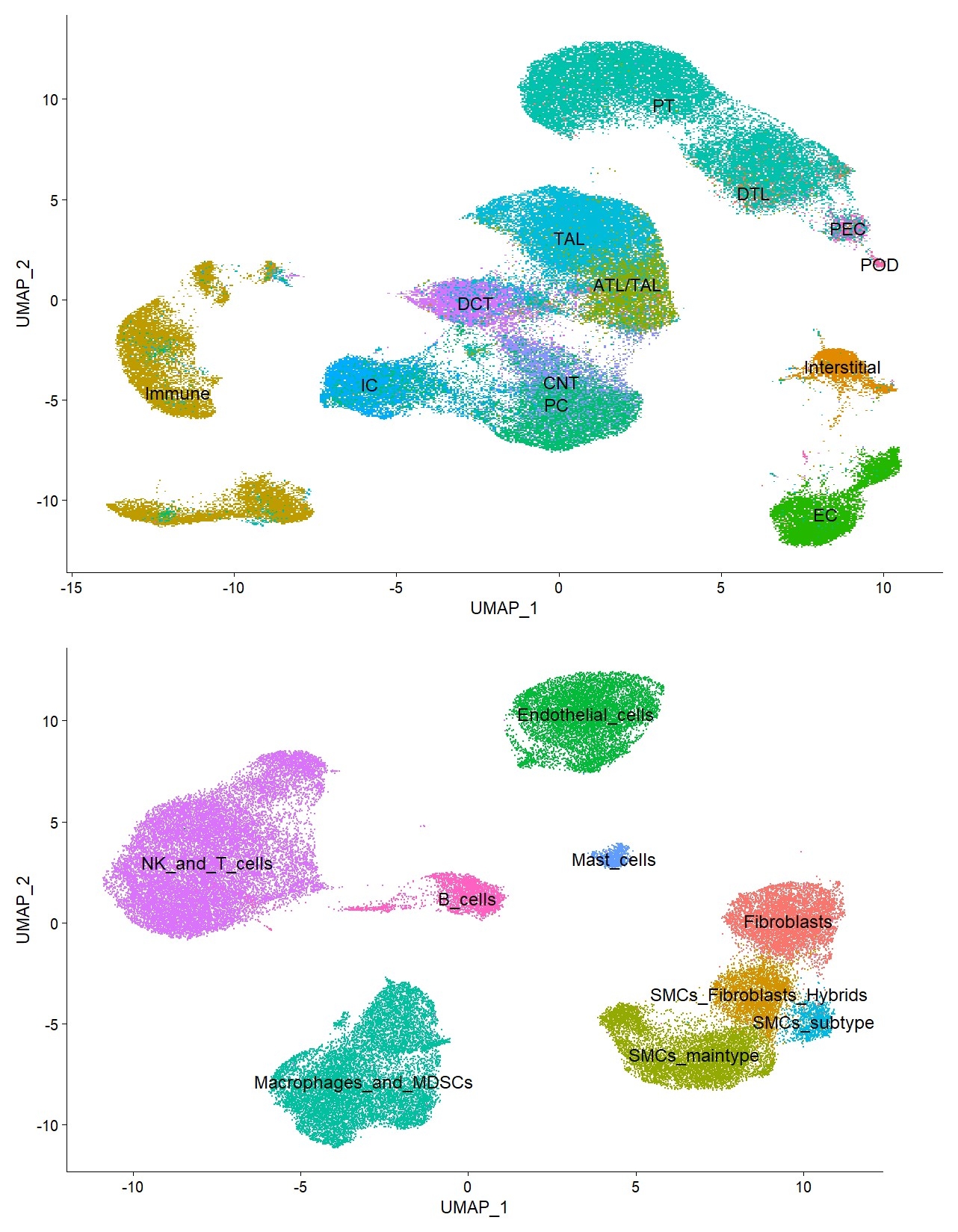

Supplement: Supplementary file 4 [file Image1.JPEG]

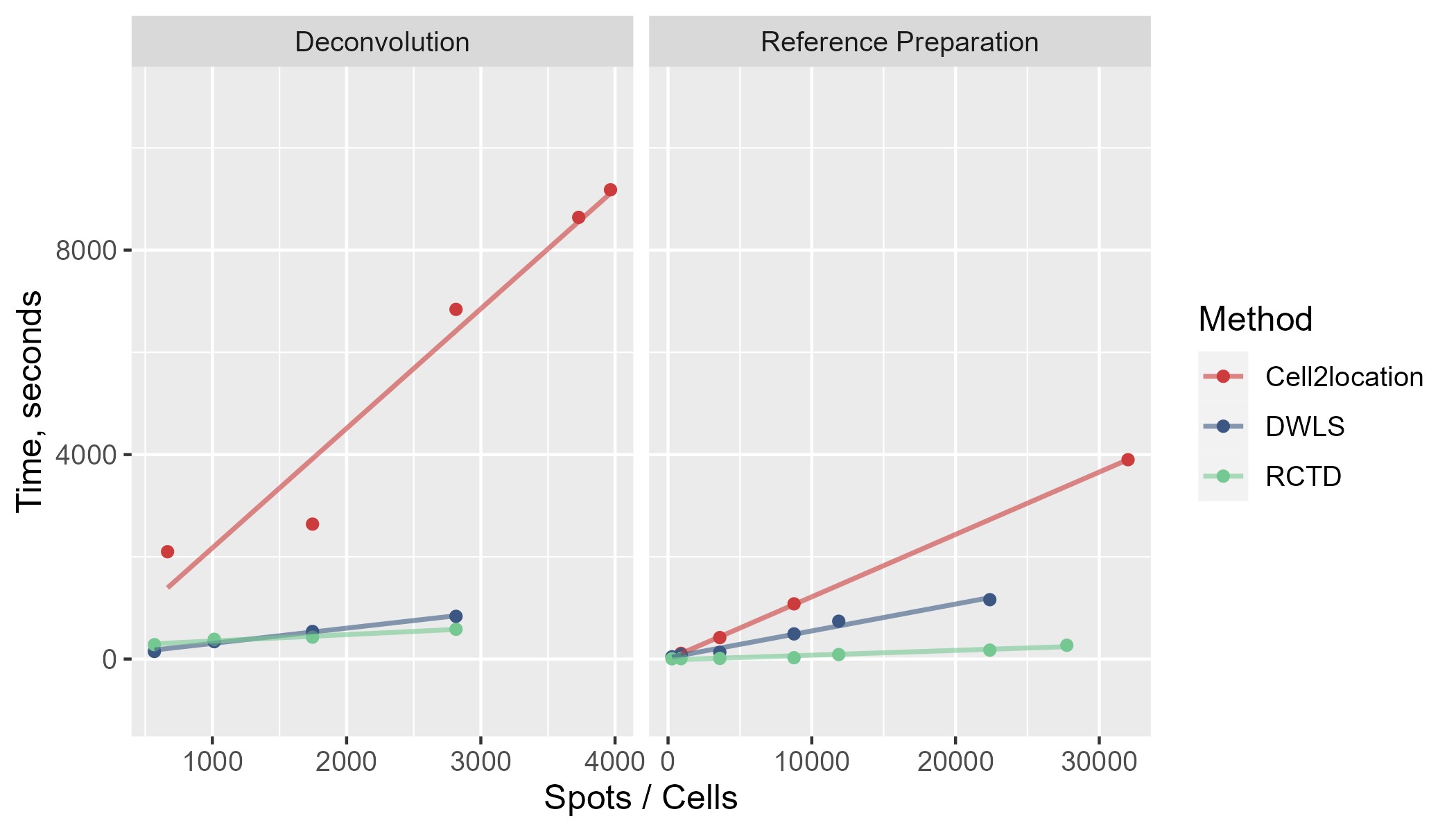

Supplement: Supplementary file 5 [file Image4.JPEG]

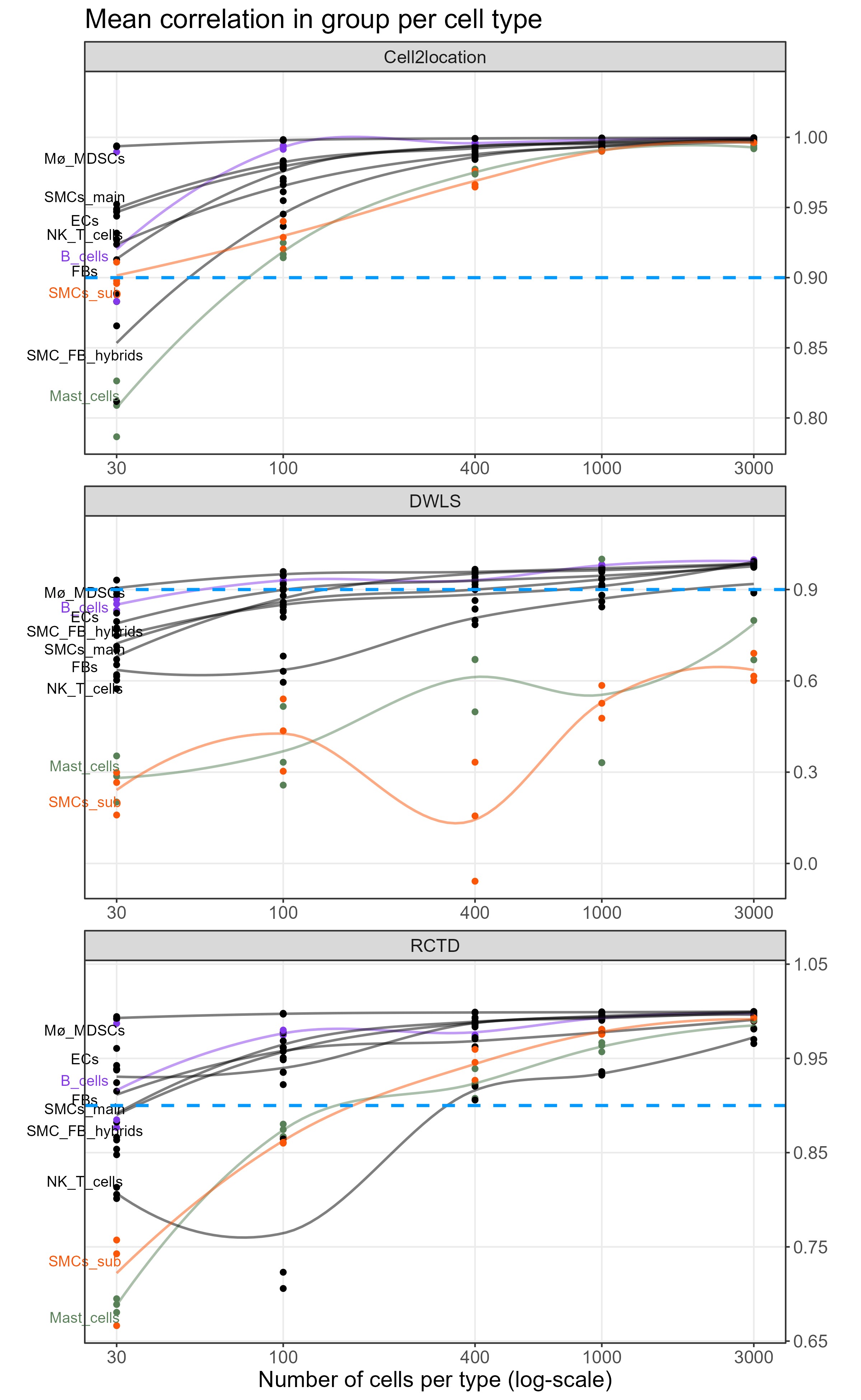

Supplement: Supplementary file 6 [file Image2.JPEG]
